# How Is Body Composition and Nutrition Status Associated with Erythropoietin Response in Hemodialyzed Patients? A Single-Center Prospective Cohort Study

**DOI:** 10.3390/jcm11092426

**Published:** 2022-04-26

**Authors:** Wiktoria Feret, Krzysztof Safranow, Kazimierz Ciechanowski, Ewa Kwiatkowska

**Affiliations:** 1 Clinical Department of Nephrology, Transplantology and Internal Medicine, Pomeranian Medical University, 70-001 Szczecin, Poland; kazcie@pum.edu.pl (K.C.); ewakwiat@gmail.com (E.K.); 2 Department of Biochemistry and Medical Chemistry, Pomeranian Medical University, 70-001 Szczecin, Poland; chrissaf@mp.pl

**Keywords:** erythropoietin resistance, body composition, hemodialysis, ERI, anemia

## Abstract

Background: Anemia is the most common finding in patients with end-stage kidney disease undergoing renal replacement therapy. A certain percentage of patients does not respond adequately to erythropoietin (EPO) treatment, not being able to reach desirable hemoglobin levels even when treated with large-dose EPO and intravenous/oral iron. In our study, we wanted to further investigate how nutritional status is associated with erythropoietin responsiveness. To quantify EPO response, we used the Erythropoietin Resistance Index (ERI), which is defined as the weekly weight-adjusted dose of EPO divided by the hemoglobin level. Patients and methods: Seventy-eight patients undergoing hemodialysis were included. All of them were measured by a SECA mBCA body composition analyzer and evaluated by Kalantar-Zadeh’s MIS score. Routine biochemical tests were also taken into account. The Shapiro-Wilk test was used to study the distributions of quantitative variables, which were significantly different from normal (*p* < 0.05). We used nonparametric Mann-Whitney U-test to compare groups. Correlations were studied by means of Spearman’s rank correlation coefficient. Bonferroni correction for multiple testing was performed. To find independent determinants of ERI, we additionally performed multivariate analysis using the General Linear Model (GLM). Results: In terms of body composition, factors that are associated with high ERI are low BMI, low fat mass, low visceral fat volume, high total body water percentage, low phase angle and low fat-free mass. In addition to body composition parameters, total MIS score and IL-6 serum levels correlated positively with ERI value. IL-6 was an independent determinant of ERI value, based on multivariate analysis. After correction for multiple analysis, BMI and eGFR both remained significant factors associated with EPO response. Conclusions: It seems crucial to prevent inflammatory malnutrition as a part of a holistic approach to anemia treatment in dialysis patients.

## 1. Introduction

Chronic kidney disease affects up to five million Poles, which constitutes 13% of the general population. Despite such a high prevalence of this disease in the population, it is still believed that the possibilities of early diagnosis and therapy are insufficient. It is estimated that about 21,000 people are currently receiving hemodialysis in Poland [1]. Hemodialysis patients must adhere to certain dietary restrictions—it is recommended that they limit their fluid intake and avoid foods that are high in sodium, potassium, and phosphate. These requirements are not easy for patients to maintain and are often associated with insufficient protein and calorie intake. Moreover, in this group of patients, depression is not uncommon, causing an additional decrease in appetite. The aforementioned difficulties contribute to the development of various types of disorders in the nutritional status of patients undergoing renal replacement therapy. However, there are factors that are beyond the patient’s control, such as the loss of amino acids and iron during each hemodialysis session [2]. Finally, the hemodialysis procedure itself increases catabolism, with a mild inflammatory reaction as an underlying cause [3]. When assessing nutritional status, most clinicians use body mass index (BMI) and classify the patients as “normal”, “overweight”, “obese” or “underweight”. Such a definition of the nutritional state may suggest that dietary intervention is sufficient to obtain clinical improvement. According to the available literature, there is another type of malnutrition, referred to as type II malnutrition: MIA syndrome (malnutrition, inflammation, atherosclerosis) or MIC (malnutrition, inflammation, cachexia); here, inflammation is a significant factor in the development of malnutrition, and the dietary intervention itself is ineffective [4,5]. Chronic inflammation can also contribute to anemia, as the expression of hepcidin increases in response to inflammation. Hepcidin is responsible for inhibiting the release of macrophage-stored iron [6]. In hemodialyzed patients, it might therefore be assumed that anemia is not only due to deficiency of erythropoietin produced and secreted in the kidneys; the mechanism is more complex. A significant proportion of patients are taking erythropoietin or other ESAs, yet the response to treatment is unsatisfactory, and ERI (erythropoietin resistance index) is notable in this group [7]. For this reason, we wanted to investigate the parameters of iron metabolism and erythropoietin response in patients undergoing hemodialysis and assess how they are associated with the nutritional status of these patients, with greater focus on body composition analysis.

## 2. Materials and Methods

This study obtained approval of the Bioethical Committee of Pomeranian Medical University in Szczecin (KB-0012/88/03/19).

The study included patients with end-stage chronic kidney disease, undergoing renal replacement therapy in The Independent Public Clinical Hospital No. 2 at Pomeranian Medical University in Szczecin. The registration period of enrolled patients was March–June 2020. All of the patients had given their informed consent to participate in the study. It consisted of three elements: blood sample collection, assessment of each individual by malnutrition inflammation score (MIS) questionnaire [8] and body composition analysis. The latter was conducted using a professional medical body composition analyzer, Seca mBCA 525, following the user manual [9]. The body weight and height of participants were both measured manually before body composition analysis. A brief description of the characteristics of main body composition elements that were measured using the Seca mBCA 252 can be found below (Table 1).

The testing is quick and non-invasive, based on an 8-point electrical impedance measurement on the patient’s body surface. The electric current used during the test is 100 µA; thus, patients with any cardiac implantable electrical device were excluded from the study, taking the measurement method into consideration. Each patient was measured after the hemodialysis procedure. The analyzer assigned each individual into one of four subsets, based on the body composition chart: increasing sarcopenic obesity, increasing obesity, increasing thinness or increasing muscle mass [9]. The authors also divided participants into four groups based on BMI: underweight (<18.5), normal (18.5–24.9), overweight (25–29.9) or obese (30 or more). Later, we compared the erythropoietin resistance index in relation to patients’ body composition chart placement and BMI as a determinant.

**Figure 1:**
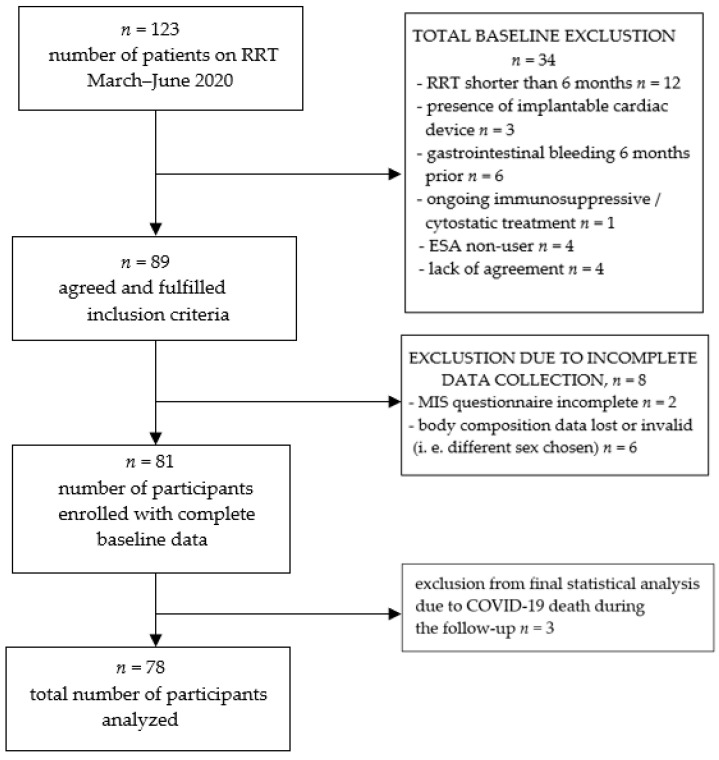
Study group recruitment.

Body surface area (BSA) was calculated separately for each individual using the Du Bois formula (BSA = 0.007184 × W^0.425^ × H^0.725^). Follow-up time was 18 months; after that, overall survival was calculated. Blood samples were collected at the baseline during routine monthly blood workup. Besides the MIS questionnaire and anthropometric measurements listed in Table 1, parameters included in the database for final analysis next to those needed to calculate ERI were age, dialysis vintage, IL-6, hepcidin, ferritin, transferrin, iron, TSAT%, PTH, eGFR, Kt/V and intradialytic weight gain (IDWG). The authors used the baseline laboratory parameters. Blood samples were drawn mid-week. The ESA preparations used among our patients were Aranesp (INN-darbepoetin alpha, by Amgen) and NeoRecormon (INN-epoetin beta, by Roche Pharmaceuticals). We recalculated the epoetin beta dose to darbepoetin alpha dose and used mean EPO units in further analysis. The erythropoietin resistance index in this study was calculated as an average weekly erythropoietin dose per kg body weight per average hemoglobin (g/dl), over the last 6 months. Taking this into account, we also excluded patients whose RRT duration was less than 6 months. EPO dosing in our center is calculated for clinically optimal body weight after dialysis. By “optimal”, the authors mean that a patient does not have any clinical symptoms of hyper- or hypovolemia (e.g., dyspnea, edemas, hypotonia, cramps, increased thirst after HD session, etc.). The flow chart of participants’ recruitment can be seen below (Figure 1).

The initial number of participants included was 81. Due to SARS-CoV2 spread during the time of the 18-month follow-up, three of them died. The authors excluded them from the final analyses, as little was known about the disease at that time. As it affected mortality in a sudden manner, the authors wanted to preserve the “natural”, previously observed mortality pattern in our group of hemodialyzed patients. Seventy-eight participants with complete data were finally taken into account in the study, 31 of which were female and 47 male. During the follow-up, there were no drop-outs due to relocation or kidney transplantation. Detailed group characteristics are given in the results section (Table 2). In our population, according to the KDIGO working definition of ESA resistance (EPO dose greater than 300 units/kg/weekly s.c. or 450 units/kg/weekly i.v. without appropriate Hgb level), only one person fulfilled these criteria. As a result, we chose ERI as a better, in our opinion, indicator of ESA response, as it takes average hemoglobin level as one of its ingredients. Similar to other researchers on EPO hyporesponsiveness, we compared groups of patients on both sides of the median and/or within terciles, as there is no clear cut-off point of ERI that defines EPO-naïve individuals by definition.

**Statistical analysis**: Statistical analysis was made using Statistica 13 software (StatSoft, Tulsa, OK, USA). The Shapiro-Wilk test was used to study the distributions of quantitative variables that were significantly different from normal (*p* < 0.05). We used nonparametric Mann–Whitney U-test to compare groups. Correlations were studied by means of Spearman’s rank correlation coefficient. Data were described as mean ± SD or median (interquartile range—IQR). *p*-values were significant when <0.05 without correction for multiple testing. Since 30 statistical tests were performed to analyze associations of ERI with other variables, the Bonferroni-corrected *p*-value threshold of significance was 0.05/30 = 0.0016. To find independent determinants of ERI, we additionally performed multivariate analysis using the General Linear Model (GLM). To assess survival, we calculated the total number of patients who died, and among those we extracted individuals who died due to cardiovascular events. Groups of survivors and deceased during the 18-month follow up were compared in terms of body composition and laboratory findings.

## 3. Results

Baseline characteristics of the study participants: Seventy-eight individuals were included in the study. The majority of them were male, with median age of 65 and median dialysis vintage of 28.5 months. Most of the participants were classified as overweight by BMI. On body composition chart, most of them were placed in the “increasing obesity” area. The median MIS score was 5. Overall 18-month mortality rate was 29.5%. Detailed laboratory findings can be found in Table 2 above.

### 3.1. Superiority of Body Composition Analysis over BMI Value in Predicting EPO Response

**Figure 2:**
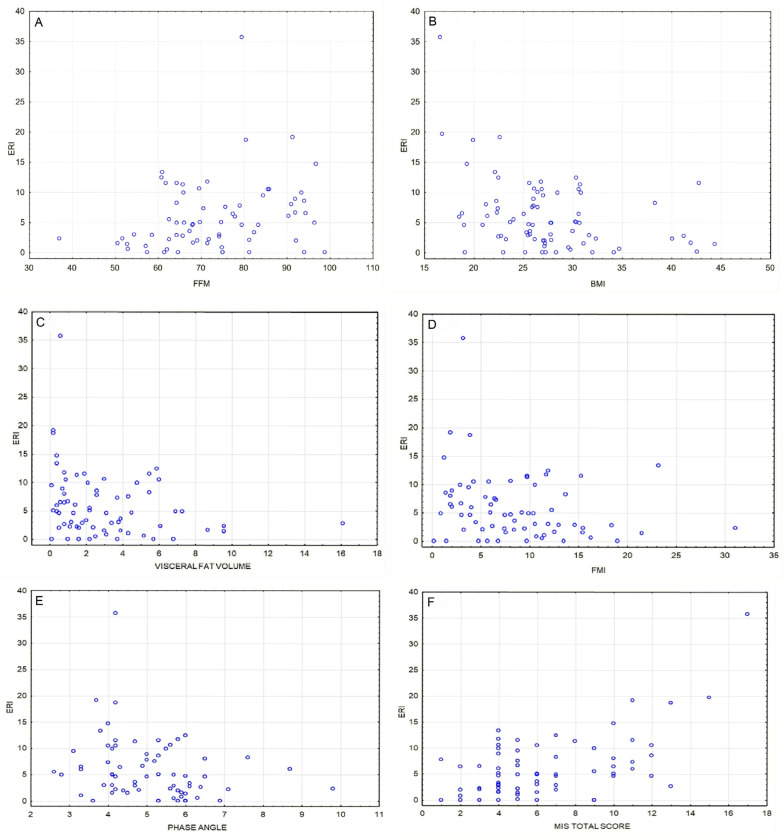
(**A**) Positive correlation between fat-free mass [%] and ERI value [IU/kg/g/dL/week] (ρ = 0.25, *p* = 0.035). (**B**) Inverse correlation between BMI [kg/m^2^] and ERI value [IU/kg/g/dL/week] (ρ = −0.33, *p* = 0.03). (**C**) Inverse correlation between visceral fat volume [l] and ERI value [IU/kg/g/dL/week] (ρ = −0.29, *p* = 0.018). (**D**) Inverse correlation between fat mass index [kg/m^2^] and ERI value [IU/kg/g/dL/week] (ρ = −0.25, *p* = 0.037). (**E**) Inverse correlation between phase angle (°) and ERI value [IU/kg/g/dL/week] (ρ = −0.33, *p* = 0.006). (**F**) Positive correlation between ERI [IU/kg/g/dL/week] and MIS total score (ρ = 0.41, *p* = 0.00041); this association remains significant after Bonferroni correction.

Whilst comparing ERI value between groups of patients categorized by BMI, median ERI was not significantly different between the groups (overweight vs. obese, normal vs. overweight and normal vs. obese), even though in general we found an inverse correlation between ERI and BMI in the Spearman’s rank test considering the whole study group (Figure 2B, see below). Body composition analysis proved to be a more precise tool in predicting erythropoietin resistance in certain cohorts, as compared groups differed significantly in terms of ERI (Table 3). Individuals that were assigned to the “sarcopenic obesity” and “obesity” group based on the body composition chart had significantly lower ERI than those in the “thinness” group. These results may suggest that adipose tissue itself is especially noteworthy when it comes to EPO resistance. The authors believe that these findings place body composition analysis over BMI as a more accurate tool in predicting erythropoietin response, as it gave statistically significant results even in a relatively small population.

In our study, we found statistically significant correlations of ERI value with certain body composition–related parameters (*p* < 0.05).

**Figure 3:**
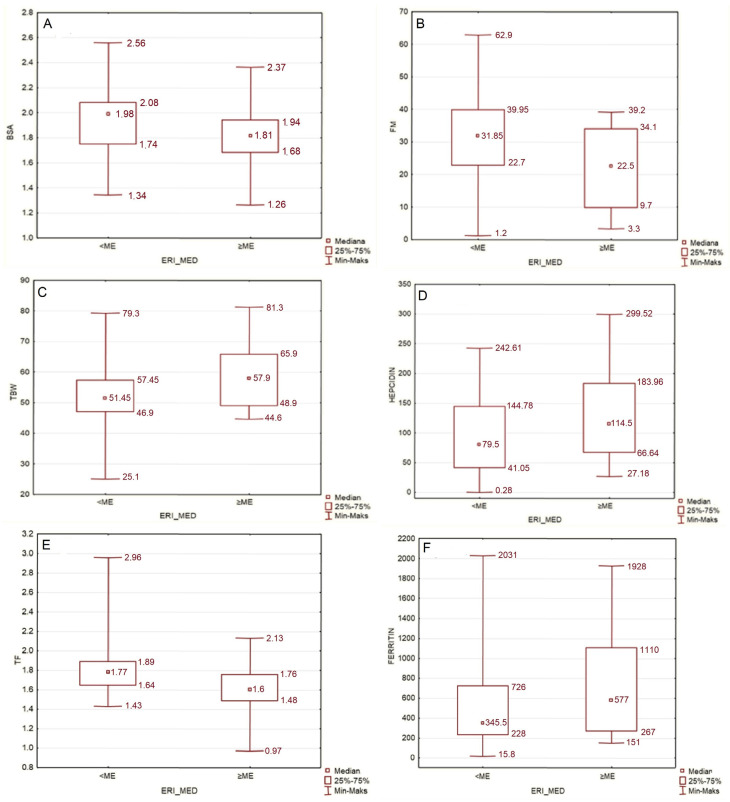
(**A**) Comparison of BSA in groups with ERI lower and higher than median (*p* = 0.033). (**B**) Comparison of FM in groups with ERI lower and higher than median (*p* = 0.024). (**C**) Comparison of TBW [%] in groups with ERI lower and higher than median (*p* = 0.024). (**D**–**F**) Comparison of hepcidin [ng/mL], transferrin [g/L] and ferritin serum level [µg/L] in groups with ERI lower and higher than median (*p* = 0.043; *p* = 0.002; *p* = 0.041, respectively).

The median ERI in our study group was 4.885. In individuals with an ERI lower than this, the median body weight was higher than in individuals whose ERI ranged above the median. The lower-ERI group, in comparison with higher-ERI group, also had higher BMI, higher BSA (Figure 3A), lower MIS score, lower fat free mass, higher fat mass (Figure 3B) and fat mass index, higher phase angle, lower total body water (Figure 3C), lower hepcidin, higher transferrin, and lower ferritin serum level (Figure 3D–F).

There was a significant, even after Bonferroni correction, inverse correlation between eGFR and ERI (ρ = −0,40; *p* = 0.0004, plot not shown).

We compared Kt/V values between groups of different body composition and BMI (Table 4), and the results were as below.

Neither parathormone serum concentration nor intradialytic weight gain (IDWG) were significantly associated with ERI in our study (ρ = −0.203; *p* = 0.076 and ρ = 0.059; *p* = 0.61, respectively; plots not shown).

### 3.2. Independent Determinants of ERI Value

To find the independent determinants of logarithmically transformed ERI, we additionally performed a multivariate analysis in which age, gender, BMI and IL-6 were independent variables. IL-6 was used as an indicator of inflammation in our study group. In multivariate analysis, low BMI and high IL-6 are factors significantly associated with high ERI, independent of age and sex; *p* = 0.003 and *p* = 0.03, respectively (Table 5, model 1).

In another model with age, gender, IL-6 and abdominal fat, and serum IL-6 and abdominal fat volume as independent variables (Table 5, model 2) low abdominal fat volume and high IL-6 concentration significantly contributed to high ERI (*p* = 0.025 and *p* = 0.0083, respectively).

### 3.3. Factors Associated with Mortality in the Study Group

After the 18-month follow-up, we calculated the total number of patients who died (*n* = 23; 29.48%), and among those, we extracted individuals who died due to a cardiovascular event (*n* = 9; 11.5% in total). We did not find statistically significant association between ERI value and all-cause mortality (*p* = 0.92) as well as death due to cardiovascular reasons (*p* = 0.1). This result, somewhat contrary to the available literature, may be caused by the relatively small sample size. Parameters we proved to be positively associated with all-cause mortality among our study group were age and MIS total score, while TBW% and serum albumin were negatively associated. In terms of death due to cardiovascular reasons, parameters we proved to be significantly positively associated were BMI, and FM, while FFM (*p* = 0.047) and TBW were negatively associated. Results are presented in the table below (Table 6).

## 4. Discussion

### 4.1. Anemia and EPO Resistance as a Major Burden in Chronic Kidney Disease

Anemia is by far the most common finding in patients with end-stage kidney disease undergoing long-term renal replacement therapy. Decreased production of native erythropoietin by the kidneys is recognized as the main cause of such a state. Taking this into account, scientists first struggled to find a way to replace the deficient hormone by its exogenous form. This led to development of the first recombinant human erythropoietin (rHuEPO), epoetin alpha, in 1989 in the US [10]. It was successfully introduced as CKD-related anemia treatment in the 1980s and has been a standard of care since then. Although other erythropoiesis-stimulating agents, including biosimilars, are gaining growing attention these days, rHuEPO is the most widely used because of its cost-effectiveness. Despite this, a certain percentage of patients seem to not respond adequately to EPO treatment, not being able to reach desirable hemoglobin levels, even when treated with large-dose EPO and intravenous iron. This fact led to further research and established a belief in the multifactorial etiology of anemia in chronic kidney disease [11,12]. Besides EPO deficiency, several aspects are nowadays known to be linked to anemia development: repetitive blood loss during each HD procedure [13,14]; functional iron deficiency linked to impaired release of iron stores from the macrophages due to hepcidin overexpression [15,16]; abundance of proinflammatory cytokines in CKD, which further leads to hepcidin overproduction [6,17,18]; impaired hematopoiesis in the bone marrow as a sequel of uremia [19]; and folate and B12 deficiency. Disturbances in all the aforementioned areas can lead not only to anemia development, but also to hemodialyzed individuals becoming EPO-naïve.

### 4.2. Erythropoietin Response and Nutrition in ESRD

It is well-established in the available literature that malnutrition carries greater mortality risk than obesity in patients undergoing renal replacement therapy, contrary to the general population [20,21]. This phenomenon is known as reverse epidemiology. In our study, we wanted to further investigate how nutritional status affects erythropoietin responsiveness. We found that high erythropoietin resistance index (ERI) in our group of hemodialyzed patients is generally related to poorer nutritional status.

In our study group, in terms of body composition analysis, individuals with poor EPO response had lower body weight, lower BMI, lower fat mass, lower visceral fat volume and lower phase angle.

#### 4.2.1. ERI and Phase Angle

A phase angle is a derivative of reactance and resistance values, obtained during bioelectrical impedance measurement [22]. It depends mainly on water and lipid content in the cell membrane and ECW/ICW ratio. Higher phase angle indicates a fair supply of nutrients and higher cell-wall stability, although there is no clear cut-off point of PhA in malnutrition/sarcopenia detection [23,24]. In poorly nourished patients, a lack of ingredients to build a stable lipo-protein component of the cell wall is the reason for low phase angle. Our findings seem to prove that sharp (low) phase angle, being a marker of cell-wall instability and fluid imbalance, can predict poor EPO response (Figure 2E). Fluid overload is also directly related to phase angle, so in HD patients, a low phase angle could indicate malnutrition, fluid overload or a combination of the two.

#### 4.2.2. ERI, Fat Mass, Fat Free Mass, Visceral Fat Volume and BMI

We found that low BMI and low fat mass in an individual were associated with higher ERI, which stands in line with the “reverse epidemiology” in CKD. Surprisingly, we also managed to find an inverse correlation between absolute visceral fat volume [l] and ERI. Whilst comparing patients assigned to one of four groups based on the body composition chart, we found that adipose tissue itself seems to be in favor of overcoming EPO resistance (Table 3). In the multivariate analysis, we found that visceral fat volume and IL-6 serum concentration are both strong predictors of EPO hyporesponsiveness (Table 2). These findings suggest that in patients with ESRD undergoing RRT, “any” kind of fatty tissue is beneficial when it comes to EPO treatment outcomes. There are many erythropoietin receptors in the adipose tissue. Although adipose tissue is known to be a source of proinflammatory cytokines, mainly IL-6, which deprives EPO sensitivity, it is also a source of leptin. Adipocyte-derived leptin has been shown to have an erythropoiesis stimulating effect. It reduces the pro-inflammatory effect of adipose tissue and enhances the anti-inflammatory effect. In an interventional study by Hung et al., high-calorie intake in HD patients leading to hyperleptinemia markedly improved hematopoiesis [25]. This is most likely why not muscle mass, but visceral fat and fat mass in general is a factor that is particularly associated with erythropoietin sensitivity [26,27].

Our research supports the results of a study by Vega et al., which showed inverse correlation between fat mass and ERI, as well as BMI and ERI [28]. Kotanko et al. studied a group of 479 African American HD patients and, similar to our research, found that higher ERI is related to low fat mass [29]. In the same study, Kotanko observed an inverse correlation between muscle mass and ERI specifically in women. In our research, higher fat-free mass was linked to higher ERI in the whole study group, independently of sex (Figure 2A–D and Figure 3B).

#### 4.2.3. ERI and Fluid Status: Total Body Water [%] and Intradialytic Weight Gain

We discovered that the total percentage of body water (TBW%) was positively correlated to ERI value. The authors hypothesize that in patients who are chronically fluid overloaded, greater hemoglobin and hematocrit levels due to hemoconcentration are less likely to be observed, and this may cause a bias on EPO-response assessment. Besides TBW%, we also investigated intradialytic weight gain (IDWG) as total body weight percentage and its relation to ERI, but in our group the correlation was not visible (ρ = 0.058; *p* = 0.61); thus, these results should be interpreted with caution. Recently, Gracia-Iguacel et al. found a link between protein-energy wasting, erythropoietin resistance and overhydration [30], concluding that patients who were overhydrated were more prone to develop protein-energy wasting. Furthermore, in the above-mentioned study, the presence of PEW was associated with higher rates of rHuEPO hyporesponsiveness. Protein-energy wasting syndrome in a hemodialyzed individual is associated with loss of muscle and fat mass due to uremia. All this stands in line with what we succeeded to show in our study: patients with low muscle and fat mass are those with higher ERI. In a small study by Hara et al., in which 14 patients on PD were enrolled, the ECW/TBW index was independently associated with ERI [31]. Although the RRT method was different in this paper, we can assume that fluid overload is linked to EPO hyporesponsiveness. Total body water (%) is linked to both fluid status and proportion of fat mass, so this association could be related to either of those factors. Fluid status and EPO response is still not well investigated and needs further studies. Nevertheless, the authors believe that insufficient control of lean body mass might play a significant role in EPO response. Taking all the above into account, repeatable body composition analysis may help achieve better clinical outcomes in patients undergoing RRT (Figure 3C).

#### 4.2.4. ERI and Malnutrition Inflammation Score

In addition to body composition analysis, we used another nutrition assessment tool: the malnutrition–inflammation score by Kalantar-Zadeh et al. [8]. This scale focuses on several elements: patient’s medical history in the last 3–6 months, physical exam regarding signs of muscle wasting and subcutaneous fat loss, BMI, and laboratory parameters such as albumin and transferrin. Total score in the MIS can range from 0 to 30 points. In our study, individuals within the highest ERI tercile also had significantly higher median MIS score than those in the lowest tercile (7 vs. 4, respectively). Our findings should be no surprise, as MIS assesses, among others, two elements that are prominent in the presence of protein-energy wasting syndrome: muscle (protein) and fat (energy) loss in an individual. As stated previously, both these factors are linked to higher ERI. Previously, some authors have proved that total MIS score correlates inversely with the severity of anemia and, on the other hand, is positively correlated with total weekly weight-adjusted dose of EPO [32,33,34,35,36] (Figure 2F).

#### 4.2.5. ERI Value and Mortality Rate

In our study, we found no link between ERI value and mortality rate, neither all-cause nor due to cardiovascular events. This stands contrary to other authors’ findings, as Lu et al. concluded that patients with higher ERI also had higher rates of all-cause mortality and cardiovascular death [37]. The same conclusions were made by Pan et al. [38]. Bae et al. found ERI value to be a predictor of all-cause mortality in hemodialysis patients, but not in peritoneal dialysis patients [39]. These differences might be due to relatively smaller sample size in comparison to the aforementioned papers, as well as shorter follow-up time; reassessment in larger, multi-center studies would be of immense value.

#### 4.2.6. ERI and IL-6 Serum Concentration

Interleukin-6 is by far one of the most important proinflammatory cytokines, with a vast spectrum of biological effects. It regulates acute phase response and acts mainly in hepatocytes, bone marrow, B-cells, T-cells and fibroblasts [40]. By stimulating hepatocytes, it promotes hepcidin production and contributes to anemia of chronic inflammation. In our study in univariate analysis, IL-6 did not correlate with the ERI value. IL-6 did not differ between terciles depending on the ERI value. As with the results of our multivariate analysis (Table 2), high IL-6 has shown to be associated with a higher ERI. In a novel clinical trial conducted by Pergola et al., lowering IL-6 level by ziltivekimab, an anti-IL-6 antibody, significantly reduced EPO requirements in HD patients [41]. Similarly, Won et al. found that hemodialyzed individuals within the highest tercile of ERI had significantly higher levels of IL-6 and concluded that IL-6 serum level is a strong predictor of poor ESA response in HD patients [42]. These findings stand in line with our study results.

#### 4.2.7. ERI and Iron-Metabolism Biomarkers: Ferritin, Transferrin and Hepcidin

We also investigated the dynamics of iron metabolism biomarkers in our study group. Patients with an ERI higher than the median, in comparison with patients with an ERI lower than the median, had higher hepcidin and ferritin serum levels as well as lower transferrin serum level. These findings can be easily explained. Hepcidin is a small protein produced by the liver, which plays a key role in iron homeostasis. It is responsible for inhibiting iron release from its storage in the macrophages and inhibits iron absorption from the gut, so its biological effect is the lowering of iron serum levels. Thus, hepcidin is expressed in any state of iron overload, such as hemochromatosis or functional iron deficit (visible in anemia of chronic diseases). Next to ferritin, it is an acute-phase protein, so concentration of both these proteins also increases in the presence of any kind of inflammation [15]. Transferrin level decreases in infection, inflammation and cachexia. These biochemical findings support the theorem that erythropoietin-resistance in ESRD is, among other factors, inflammation-driven (Figure 3D–F).

#### 4.2.8. ERI and Dialysis Adequacy: Kt/V and the Effect of Uremia on EPO Response

Inadequate dialysis is thought to play an important role in erythropoietin response, as the concentration of uremic toxins leads to chronic activation of the inflammatory cascade and various metabolic consequences, with blunted erythropoiesis being one of them. The most commonly used indicator of dialysis adequacy is Kt/V_urea_, which is a dimensionless measure of urea removal during a single RRT procedure. In this ratio, K stands for dialyzer clearance and represents the volume of blood in milliliters that passes through the dialyzer per minute. K is specific for the dialyzer model. Another factor, *t*, indicates dialysis time in minutes, and V in the denominator represents the volume distribution of urea. It is believed in everyday practice that with greater values of Kt/V, dialysis is more effective. Nevertheless, we should bear in mind that urea, although best known, is not the only toxic molecule that needs to be removed during dialysis: The European Uremic Toxin Work Group described over 140 substances that have a negative impact on biological functions while not excreted by the kidneys [43]. Developing different membranes, dialysis fluids and HD techniques with greater biocompatibility allows clinicians to slightly impede the burden of chronic uremic inflammation. Still, the way to achieve high dialysis adequacy in an individual varies globally, depending on the region’s income and public health policy [44]. Previously published studies proved that adequate dialysis (measured by Kt/V) allowed lower rHuEPO doses. Having found this, we further compared Kt/V indexes among groups of different body types based on BMI and BCA (Table 4). In our cohort, obese individuals had lower Kt/V than those classified as “normal” by BMI and “thin” by BCA. These results unfortunately indicate that in our dialysis center, RRT quality for obese patients is not relevant. Hruby and Nowicki suggested that for individuals with greater body weight, better effects can be achieved by increasing blood flow and dialyzer surface [45,46]. Regrettably, our dialysis center does not have access to high-flux dialyzers, so the healthcare policy in our region negatively impacts treatment results and may be a cause of such bias in this study; for patients with lower BMI, our standard low-flux dialyzer may be enough, and the dialyzer surface will also be adequate, but it is insufficient for obese patients. On the other hand, our obese group still had lower ERI despite lower Kt/V. Our results considering ERI and Kt/V should therefore be interpreted with caution. Many interesting reports have recently suggested that Kt/V is an obsolete indicator of dialysis adequacy, as it does not take individual patient characteristics (e.g., “other-than-average” body composition) and other uremic solutes into account [47,48,49]. It is undoubtable that accumulation of other uremic toxins (such as beta-2-microglobulin, IL-6, indoxyl sulfate, *p*-cresyl sulfate, etc.) and not urea alone, accounts for dysregulation of erythropoiesis and malnutrition aggravation in hemodialyzed patients through a variety of mechanisms that are beyond the range of this publication [49,50].

## 5. Conclusions

Screening for the possible underlying reasons of EPO hyporesponsiveness in a hemodialyzed individual does not require great effort and can be made using simple tools, such as body composition analysis and/or MIS paired with IL-6 serum level testing. Our study further established some previously observed patterns, that generally malnourished patients with chronic inflammation are more prone to develop EPO resistance. The most crucial take-home point from our study is, in our opinion, the importance of adipose tissue in overcoming erythropoietin resistance. Adipocyte-derived leptin is believed to stimulate erythropoiesis and dampen the pro-inflammatory effect of visceral obesity in terms of red blood cell production. Bearing all this in mind, it seems crucial to prevent malnutrition and frailty as a part of a holistic approach to anemia treatment in dialysis patients. Clinicians should take an individualized approach towards hemodialysis techniques for each patient and cooperate with nutritionists and physiotherapists to ensure adequate macro- and micronutrient dietary intake, prevent fatty tissue loss, control fluid overload, reduce oxidative stress and prevent sarcopenia through patient-adjusted physical activity.

## 6. Strengths

The study group was assessed not only with a nutritional questionnaire, but also with examination of body composition. Erythropoietin resistance was calculated over 6 months of treatment. Follow-up to assess overall mortality lasted 18 months.

## 7. Limitations

This study involved a relatively small sample size and was a single-center study. Only two associations of ERI (positive with and MIS total score and negative with eGFR) remained significant after Bonferroni correction for multiple testing.

## Figures and Tables

**Table 1:** List of measured body composition parameters.

Parameter	Description
BMI—body mass index [kg/m^2^]	A value derived from body mass divided by the square of the body height, traditionally used to group individuals as underweight, normal, overweight or obese.
FFM—fat free mass [kg], relative to weight [%]	Calculated by subtracting body fat weight from total body weight; also referred to as “lean body mass”.
FFMI—fat free mass index [kg/m^2^]	Describes the amount of fat-free mass (“lean body mass”) in relation to height and weight. Similar to BMI.
FM—fat mass [kg], relative to weight [%]	Total amount of fat; percentage of total bodyweight that is fat.
FMI—fat mass index [kg/m^2^]	Describes the amount of fat mass in relation to height and weight. Similar to BMI.
TBW—total body water [l], relative to weight [%]	The sum of intracellular water and extracellular water volume; approx. 60% of body weight of a normovolemic individual.
Phase angle φ [°]	Calculated by reactance/resistance ratio during bioelectrical impedance measurement. Used as an indicator of cell wall stability. Helpful in health risk assessment.
VAT—visceral adipose tissue [l]	Also known as abdominal fat, describes adipose tissue that surrounds the organs in the abdominal cavity. Overdeposition of visceral fat in the abdomen is known as visceral obesity.

**Table 2:** Group characteristics.

Overall Participants	*n* = 78	
Male	*n* = 47 (60.3%)	
Age [years]	Median: 65; IQR = 21	
Dialysis vintage [months]	Median: 28.5; IQR = 42	
Patients’ nutrition by BMI [%]	underweight	2.6%
	normal	26.9%
	overweight	42.3%
	obese	28.2%
Patients’ nutrition by SECA mBCA body composition chart [%]	increasing sarcopenic obesity: 23.2% increasing obesity: 30.4% increasing thinness: 17.4% increasing muscle mass: 29%	
ERI [IU/kg/g/dL/week]	Median: 4.9; IQR = 6.8	
IL-6 [pg/mL]	Median: 3; IQR = 2.9	
Albumin [mg/mL]	Median: 41; IQR = 5	
Transferrin [g/L]	Median: 1.7; IQR = 0.26	
Transferrin saturation [%]	Mean: 29.2 (SD 12.7)	
Hepcidin [ng/mL]	Median: 92.55; IQR = 108.8	
Ferritin [µg/L]	Median: 475; IQR = 557	
Hemoglobin [mmol/L]	Mean: 6.72 (SD 0.86)	
PTH [pg/mL]	Median: 322; IQR = 290	
Kt/V	Mean: 1.14 (SD 0.23)	
Intradialytic weight gain [% of total body weight]	Median: 2.26; IQR = 2.82	
eGFR [mL/min/1.73 m^2^]	Median: 7; IQR = 4	
Total MIS score	Median: 5; IQR = 5	
Mortality rate (18-month follow-up)	Overall: *n* = 23 (29.5%) Cardiovascular reasons: *n* = 9 (11.5%)	

Abbreviations: IQR—interquartile range, BMI—body mass index, mBCA—medical body composition analyzer, ERI—erythropoietin resistance index, eGFR—estimated glomerular filtration rate, MIS—malnutrition inflammation scale.

**Table 3:** BMI vs. BCA as predictors of ESA response (significant results in bold, *p* < 0.05).

Comparison of BMI and mBCA as Predictors of ERI Value	
**BMI Group**	**ERI, Median; IQR**
underweight	not included in the statistical analysis due to small sample size (*n = 2*)
normal	6.1; 4
overweight	3.5; 5.8
obese	3.2; 6.7
***Comparison of BMI groups (U-Mann-Whitney Test)***	
*ERI overweight* vs. *obese*	*p* = 1
*ERI normal* vs. *overweight*	*p* = 0.09
*ERI in normal* vs. *obese*	*p* = 0.1
**BCA Group**	**ERI, Median; IQR**
increasing sarcopenic obesity	2.8; 4.2
increasing obesity	2.9; 6.7
increasing thinness	6.01; 8.03
increasing muscle mass	6.5; 7.2
***Comparison of BCA Groups (U-Mann-Whitney Test)***	
*ERI sarcopenic obesity* vs. *obesity*	*p* = 0.8
*ERI sarcopenic obesity* vs. *thinness*	*p* = 0.02
*ERI sarcopenic obesity* vs. *muscle mass*	*p* = 0.52
*ERI obesity* vs. *thinness*	*p* = 0.02

**Table 4:** Kt/V and nutrition as determined by BMI and body composition analysis.

KT/V and Nutrition Comparison between Groups (U-Mann-Whitney Test)			
In Groups Divided by BMI		In Groups Divided by BCA	
Category	KT/V, Mean; SD	Category	KT/V, Mean; SD
normal	1.24; 0.24	increasing sarcopenic obesity	1.1; 0.23
overweight	1.13; 0.20	increasing obesity	1.1; 0.21
obese	1.05; 0.21	increasing thinness	1.31; 0.30
		increasing muscle mass	1.12; 0.15
*normal* vs. *overweight*	*p* = 0.10	*sarcopenic obesity* vs. *obesity*	*p* = 0.72
*normal* vs. *obese*	*p* = 0.02	*sarcopenic obesity* vs. *thinness*	*p* = 0.052
*overweight* vs. *obese*	*p* = 0.41	*sarcopenic obesity* vs. *muscle mass*	*p* = 0.77
		*obesity* vs. *thinness*	*p* = 0.02
		*obesity* vs. *muscle mass*	*p* = 0.55
		*thinness* vs. *muscle mass*	*p* = 0.07

**Table 5:** General Linear Model (GLM) analysis of independent determinants of ERI value in terms of body composition and inflammatory indicators. (VFT—visceral fat tissue, IL-6—interleukin-6, BMI—body mass index).

**GLM MODEL 1 (*p* = 0.0069)**				
	**Beta (ß)**	**−95%CI Beta**	**+95%CI Beta**	***p***
SEX	−0.037	−0.26	0.18	0.73
AGE	−0.038	−0.26	0.19	0.74
BMI	−0.34	−0.56	−0.12	0.003
Log IL-6	0.25	0.026	0.47	0.03
**GLM MODEL 2 (*p* = 0.016)**				
	**Beta (ß)**	**−95%CI Beta**	**+95%CI Beta**	***p***
SEX	0.11	−0.14	0.35	0.4
AGE	−0.04	−0.28	0.21	0.76
Log VFT	−0.35	−0.6	−0.093	0.0083
Log IL-6	0.27	0.034	0.5	0.025

**Table 6:** Comparison of survivors and deceased in terms of ERI, body composition, dialysis vintage and significant laboratory findings (U-Mann-Whitney test, IQR—interquartile range).

**Death of Any Cause**			
	**Deceased (*n* = 23)**	**Survivors (*n* = 55)**	***p*-Value**
ERI value (median; IQR)	4.98 (7.02)	4.88 (7.71)	*p* = 0.92
Age, years (mean)	69.7	59.6	*p* = 0.0069
MIS total score (median; IQR)	9 (6.5)	5 (3)	*p* = 0.00087
TBW, % (median; IQR)	49.3 (8)	55.9 (14.5)	*p* = 0.029
Serum albumin (median; IQR)	38.5 (4)	42 (5)	*p* = 0.00034
Dialysis vintage in months (median, IQR)	32 (37)	25 (45)	*p* = 0.81
**Death Due to Cardiovascular Disease**			
	**Deceased (*n* = 9)**	**Survivors (*n* = 55)**	***p*-Value**
ERI value (median; IQR)	1.35 (4.53)	4.96 (7.2)	*p* = 0.1
BMI, [kg/m^2^] (median; IQR)	29.77 (11.44)	26.16 (7.35)	*p* = 0.04
FFM, % (median; IQR)	63.2 (14.1)	74.3 (18.7)	*p* = 0.047
FM, % (median; IQR)	36.8 (14.1)	25.7 (18.7)	*p* = 0.047
TBW, % (median; IQR)	47 (6.75)	55.4 (12.2)	*p* = 0.0051
Dialysis vintage in months (median, IQR)	28 (56)	29 (36)	*p* = 0.66

## Data Availability

For additional data, please contact ewakwiat@gmail.com.
